# Azidothymidine Sensitizes Primary Effusion Lymphoma Cells to Kaposi Sarcoma-Associated Herpesvirus-Specific CD4+ T Cell Control and Inhibits vIRF3 Function

**DOI:** 10.1371/journal.ppat.1006042

**Published:** 2016-11-28

**Authors:** Samantha J. Williamson, Samantha M. Nicol, Michael Stürzl, Shereen Sabbah, Andrew D. Hislop

**Affiliations:** 1 Institute of Immunology and Immunotherapy, University of Birmingham, Edgbaston, Birmingham, United Kingdom; 2 Division of Molecular and Experimental Surgery, Department of Surgery, Friedrich-Alexander-University Erlangen-Nürnberg, Erlangen, Germany; 3 Department of Immunobiology, King's College London, London, United Kingdom; University of North Carolina at Chapel Hill, UNITED STATES

## Abstract

Kaposi sarcoma-associated herpesvirus (KSHV) is linked with the development of Kaposi sarcoma and the B lymphocyte disorders primary effusion lymphoma (PEL) and multi-centric Castleman disease. T cell immunity limits KSHV infection and disease, however the virus employs multiple mechanisms to inhibit efficient control by these effectors. Thus KSHV-specific CD4+ T cells poorly recognize most PEL cells and even where they can, they are unable to kill them. To make KSHV-infected cells more sensitive to T cell control we treated PEL cells with the thymidine analogue azidothymidine (AZT), which sensitizes PEL lines to Fas-ligand and TRAIL challenge; effector mechanisms which T cells use. PELs co-cultured with KSHV-specific CD4+ T cells in the absence of AZT showed no control of PEL outgrowth. However in the presence of AZT PEL outgrowth was controlled in an MHC-restricted manner. To investigate how AZT sensitizes PELs to immune control we first examined BJAB cells transduced with individual KSHV-latent genes for their ability to resist apoptosis mediated by stimuli delivered through Fas and TRAIL receptors. This showed that in addition to the previously described vFLIP protein, expression of vIRF3 also inhibited apoptosis delivered by these stimuli. Importantly vIRF3 mediated protection from these apoptotic stimuli was inhibited in the presence of AZT as was a second vIRF3 associated phenotype, the downregulation of surface MHC class II. Although both vFLIP and vIRF3 are expressed in PELs, we propose that inhibiting vIRF3 function with AZT may be sufficient to restore T cell control of these tumor cells.

## Author Summary

Kaposi sarcoma-associated herpesvirus (KSHV) can cause disease in humans in the form of B lymphocyte disorders such as primary effusion lymphoma (PEL) and multicentric Castleman disease. Where tested, these are highly resistant to immune control by KSHV-specific T cells. To investigate how such KSHV-infected cells can be made more sensitive to T cell control we treated PEL lines with azidothymidine (AZT), which has been shown to induce sensitivity in such lines to the mechanisms which T cells use to kill targets. We found this allowed the T cells to control in vitro lymphoma growth. The ability of the T cells to control PEL cell growth was found to correlate with AZT mediated inhibition of function of the KSHV protein vIRF3 which we show has the ability to protect cells from killing by immune effector mechanisms. These studies suggest that the therapeutic drug AZT may be of use to tip the virus host balance away from the virus by interfering with this immune evasion and pro-survival protein, potentially allowing better control by the host.

## Introduction

Kaposi sarcoma-associated herpesvirus (KSHV) is an oncogenic human γ-herpesvirus which infects endothelial cells and establishes a latent infection in B lymphocytes. It is associated with the endothelial cell malignancy Kaposi sarcoma (KS) and the B lymphocyte disorders primary effusion lymphoma (PEL) and multi-centric Castleman disease (MCD)[1]. The immune response is important in controlling infection and disease caused by KSHV as seen by the higher frequency of KSHV-associated disease found in immunosuppressed patients such as HIV or transplant patients[2]. Restoration of immune competence in KS patients can lead to resolution of this malignancy [3,4], implying an important role for the cellular immune response in the control of KSHV-infection and disease.

To control KSHV-malignancies the cellular immune response must overcome immune evasion mechanisms employed by the virus. These include the production of a restricted repertoire of proteins, limiting the range of immune targets but allowing establishment of a predominantly latent non-virus productive infection. Proteins expressed within KSHV malignancies include the genome maintenance protein LANA, a cyclin D homologue vCyclin, an NF-κB activator with pro-survival function vFLIP, and the Kaposin proteins of which the best characterized, Kaposin B, functions to stabilize cytokine mRNAs (for review see Schulz and Cesarman[5]). Some of these genes show intrinsic features which likely minimize exposure to CD8+ T cells by restricting synthesis of their encoded protein, reducing the supply of defective ribosomal products (DRiPs) that are thought to be the source of CD8+ T cell peptide-epitopes[6]. Firstly vFLIP utilizes inefficient codons resulting in the production of unstable mRNA and low levels of protein expression[7]. Secondly LANA encodes extensive repeat sequences which restrict translation and proteasomal mediated destruction[8], minimizing epitope presentation from this protein to CD8+ T cells[9]. KSHV B cell pathologies additionally express an interleukin-6 homologue vIL-6, and the multifunctional protein vIRF3. Amongst other functions, vIRF3 can inhibit p53 and IRF5 function[10,11] as well as decrease surface MHC class II expression through inhibiting the promoter of the class II transcriptional transactivator CIITA[12]. Additionally, infected B cells express the ubiquitin ligases K3 and K5, which induce endocytosis of surface MHC class I and co-stimulatory molecules such as ICAM and CD86[13,14]. These multiple layers of immune evasion mechanisms represent a challenge for T cell mediated control of infected cells.

Studies using CD8+ T cells to probe recognition of PELs expressing reporter antigens have shown that they were unable to recognize these targets[15]. Recognition of PELs by LANA-specific CD4+ T cells, which would be less affected by the restricted production of this protein as CD4 epitope generation is not reliant on the DRiP pathway, is also mostly poor[16]. This is likely due to vIRF3 expression as PELs which either constitutively[16] or transiently[17] express reduced levels of vIRF3 have increased levels of surface MHC class II and can be recognized by the T cells. However in these cases or when vIRF3 function is bypassed and expression of class II is restored to allow recognition of PELs by LANA-specific CD4+ T cells, these effectors are not able to kill PELs despite killing other target cell types in parallel assays[16]. Consistent with this finding is that PEL lines are highly resistant to cell death induced by effector mechanisms that T cells employ, namely stimulation via the extrinsic apoptotic pathways through Fas or TNF-related apoptosis inducing ligand (TRAIL) receptors[18,19].

The finding that knock down of vFLIP induced sensitivity to Fas ligation indicates that this viral protein can interfere with this apoptotic pathway[18], however vFLIP is thought to be expressed at low levels within infected cells[7]; whether other viral proteins contribute to this inhibition is unclear. An understanding of whether other latent proteins inhibit pathways induced by extrinsic apoptotic stimuli would allow the rational design of interventions to inhibit these functions and restore sensitivity to T cell effector mechanisms. Nevertheless some chemotherapeutic approaches to increase sensitivity of PEL cells to immune clearance have been developed. In particular, treating PEL cell lines with the thymidine analogue azidothymidine (AZT) renders them sensitive to Fas-ligand or TRAIL mediated killing [19,20]. Additionally AZT has been used therapeutically in combination with IFNα to induce TRAIL expression in PELs, resulting in their apoptosis [21]. AZT treatment of PELs induces some cleavage of caspase 3 [19] and its mechanism of action has been linked to reducing nuclear translocation of NF-κB. Specifically the mono-phosphorylated form of AZT has been found to inhibit in vitro IKKβ-mediated phosphorylation of the NF-κB regulator IκBα [21]. However compared to other NF-kB inhibitors AZT has a more subtle effect on PELs, not obviously altering growth or apoptosis, while classic inhibitors such as BAY 11–7082 or Bortezomib rapidly induce apoptosis[22,23].

As T cells are likely to express Fas-ligand or TRAIL, we determined whether KSHV-specific CD4+ T cells could inhibit the outgrowth of MHC matched AZT-treated PEL lines and found that this treatment induced sensitivity of PELs to T cell control. We also identified that in addition to vFLIP, expression of vIRF3 prevented apoptosis induced by extrinsic pathways and that AZT treatment inhibited this vIRF3 mediated protection.

## Results

### AZT treatment of PEL cells induces sensitivity to KSHV-specific CD4+ T cells

We have previously found that KSHV-specific CD4+ T cells poorly recognize most MHC-matched PEL cell lines, as few PEL cells only transiently express surface MHC class II at any one time [17]. Engineering PELs to express surface MHC class II allowed recognition but not killing by the T cells, despite these effectors killing other targets in parallel assays [16]. Culturing PELs in the thymidine analogue azidothymidine (AZT) induces sensitivity to apoptosis mediated by Fas-ligand and TRAIL [19,20] and as these can be expressed by T cells, we asked whether AZT would sensitize PELs to control by KSHV-specific CD4+ T cells.

We initially determined whether a panel of established KSHV-specific CD4+ T cell clones [16] (Table 1) expressed Fas-ligand and TRAIL transcripts by qRT-PCR analysis after stimulation with autologous target cells sensitized with their cognate peptide-epitope. Transcript expression was estimated and the levels detected shown in Table 1, expressed relative to those seen in peripheral blood mononuclear cells (PBMC) activated with phorbol myristate acetate and ionomycin to induce transcription of these genes[24,25]. Compared to activated PBMC, the clones expressed Fas-ligand or TRAIL transcripts in most cases at similar levels to activated PBMC.

**Table 1:** Characteristics of KSHV-specific CD4+ T cells.

Protein	Amino acid location*	Peptide sequence	Restriction	Functional avidity (M)	TRAIL expression^†^	Fas ligand expression^†^
LANA	191–205	**LAP**STLRSLRKRRLS	DP1	10^−8^	0.83	0.83
LANA	196–210	**LRS**LRKRRLSSPQGP	DR13	10^−6^	1.11	1.46
LANA	191–210	**LAP**STLRSLRKRRLS/**LRS**LRKRRLSSPQGP	DQ6	10^−8^	3.57	0.28
LANA	66–80	**GSP**TVFTSGLPAFVS	DQ7	10^−7^	0.1	0.77
vCyclin	41–55	**TFQ**QSLTSHMRKLLG	DQ6	10^−7^	0.78	1.23

*Coordinate location based on BC-1 KSHV sequences

†Transcript level expressed relative to activated PBMC

**Fig 1:**
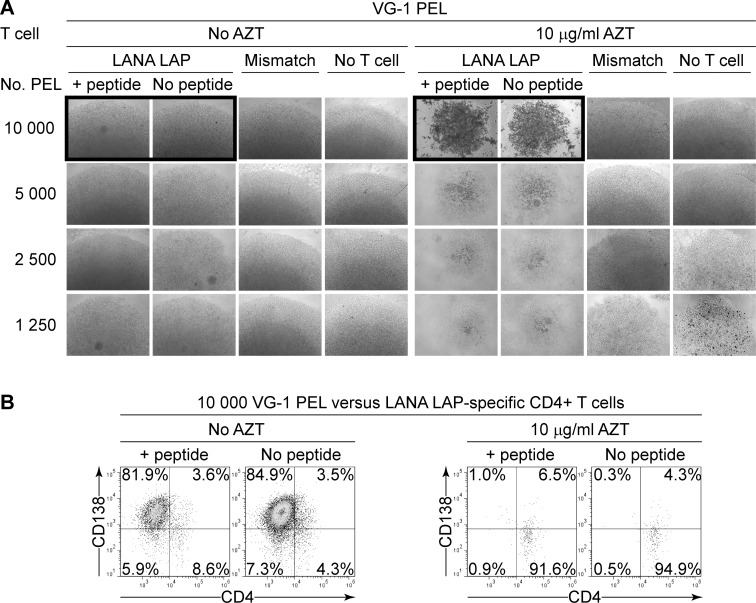
Outgrowth assays of PEL cells challenged with KSHV-specific T cells in the presence or absence of AZT. (A). VG-1 PELs which had been treated with 10 μg/ml AZT for at least one week were either pre-sensitized with LAP-peptide or not and seeded in triplicate cultures at doubling dilutions from 10^4^ cells per well to 1 250 cells per well. To these, 10^4^ MHC-matched LAP-specific CD4+ T cells or mismatched CD4+ T cell clones were added and, where indicated, AZT added to a final concentration of 10 μg/ml. Cultures were allowed to grow for 10 days and cell pellets then visualized by microscopy. Images show representative cultures of triplicate wells. (B). Flow cytometric phenotyping of cell populations present at day 10. Cells from the wells highlighted with the black outline in Fig 1A were stained for expression of CD138 or CD4 and analyzed by flow cytometry.

We next developed an in vitro assay to test the ability of KSHV-specific CD4+ T cells to control outgrowth of MHC-matched AZT-treated PEL lines. Here T cells were co-cultured with PELs for ten days so all PELs would likely transiently express MHC class II at some time during the assay, allowing T cell recognition. PELs were grown in 10 μg/ml AZT, which did not lead to any consistent changes in latent gene expression across the PELs as judged by qRT-PCR analysis (S1A Fig). Additionally little if any inhibition of proliferation of PELs was seen when cultured in the presence of AZT (S1B Fig) as previously described [19]. Doubling dilutions from 10 000 PELs were seeded as triplicate microcultures in 96 well plates and 10 000 MHC-matched KSHV-specific CD4+ T cells were added to each replicate. To act as controls PELs were either sensitized with the T cells cognate peptide-epitope, or challenged with MHC-mismatched T cells to assess non-specific inhibition of PEL growth. Additionally, dilutions of PELs were seeded in the absence of T cells to monitor any growth inhibition from AZT. Identity of the outgrowing cells was determined by flow cytometry analysis, staining for CD138 and CD4 expression as markers of PELs or T cells respectively. Fig 1 shows representative results of an assay using the PEL VG-1 and DP-1 restricted, LANA-encoded LAPSTLRSLRKRRLS-specific CD4+ T cells; T cells are subsequently identified by the first three amino acids of their cognate peptide-epitope. Microscopic images presented in Fig 1A show representative results of assays conducted and indicate that there was substantial growth of cells under some conditions but not others. Thus PELs cultured in the absence of T cells showed little or no outgrowth inhibition in the presence or absence of AZT. Co-cultures of PELs with T cells showed that in the absence of AZT there was substantial cell growth; it would seem unlikely that the cells proliferating here would be the T cell clones as these require high levels of cytokines to proliferate. Indeed flow cytometry analysis of these populations presented in Fig 1B (left hand panels) confirmed that they were dominated by CD138 positive PEL cells and that no additional control of outgrowth was seen when PELs had been sensitized with epitope-peptide. Parallel assays conducted in the presence of 10 μg/ml AZT, as shown in Fig 1A (right hand panels), showed cell outgrowth when MHC-mismatched T cells were co-cultured with PELs. However no obvious outgrowth of cells was seen in co-cultures of VG-1 with MHC-matched LAP-specific T cells in the presence of AZT; furthermore flow cytometry analysis confirmed that only CD4+ T cells could be detected in these wells (Fig 1B right hand panels). Importantly, PEL outgrowth was controlled even when they were not sensitized with the peptide-epitope, implying there was sufficient recognition of the endogenously processed and presented epitope to allow control.

**Fig 2:**
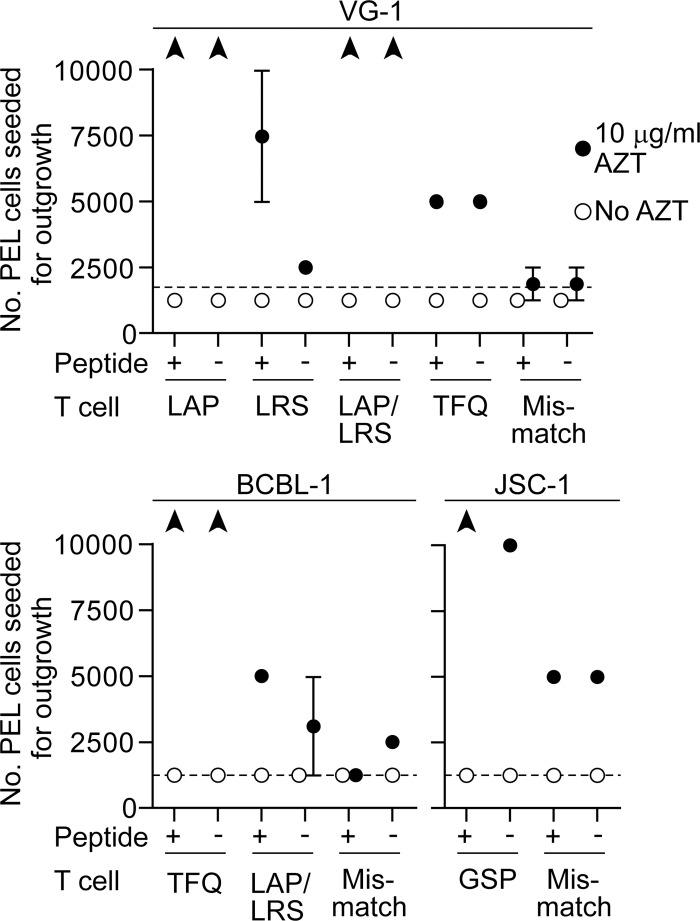
Summaries of PEL outgrowth assays after challenge with KSHV-specific CD4+ T cells in the presence or absence of 10 μg/ml AZT. Cell outgrowth at day 10 was scored after confirming cell identity by flow cytometry for the indicated PEL and T cell combinations. Data is expressed as the minimum number of PELs seeded which successfully outgrew the T cells and the dashed line represents the number of AZT treated PELs seeded in the absence of T cells to achieve outgrowth. Results shown are averages from at least two assays with error bars representing the standard error of the mean. Black arrowheads indicate greater than 10^4^ PELs were required to outgrow the T cells.

Fig 2 shows averaged results of outgrowth assays graphed as the number PEL cells seeded required to outgrow T cells. Here the PEL cell lines VG-1, BCBL-1 and JSC-1 were assayed on at least two occasions with a total of five different MHC-matched CD4+ T cell clones specific for vCyclin or LANA epitopes. In all cases, assays conducted without AZT showed no T cell control of PEL outgrowth, regardless of whether the PELs had been pre-sensitized with the T cell’s peptide-epitope. However parallel assays using MHC-matched T cell clones showed that in the presence of 10 μg/ml AZT, PEL growth could be inhibited and in some cases completely controlled. By contrast, culturing MHC-mismatched T cell clones with AZT treated JSC-1 showed some control of these PELs while VG-1 or BCBL-1 showed little or no growth inhibition when cultured with mismatched clones indicating the specificity of T cell control. Of note, the inhibition of outgrowth appeared relatively slow. Control of PEL outgrowth took several days, even in the case of BCBL-1 where all cells expressed surface class II. Thus VG-1 cells cultured with AZT assayed versus LAP- or LAP/LRS-specific clones were ultimately completely controlled by these effectors, while TFQ-specific cells showed increased control over background. LRS-specific clones showed the lowest increase in control of non-peptide sensitized VG-1 cells with AZT compared to the MHC-mismatch cells which may reflect their lower functional avidity (10^−6^ M; Table 1) however sensitizing the PELs with LRS peptide increased control by these T cells. Similar results were observed using the BCBL-1 and JSC-1 PELs, assayed against appropriately matched T cells. Additionally we confirmed that T cells pre-treated with AZT showed equivalent control in outgrowth assays (S2 Fig). These findings indicate that most KSHV-specific T cells recognize sufficient epitope derived from the endogenously expressed protein to induce effector function in these longer term assays and that AZT sensitizes PEL cells to T cell control.

**Fig 3:**
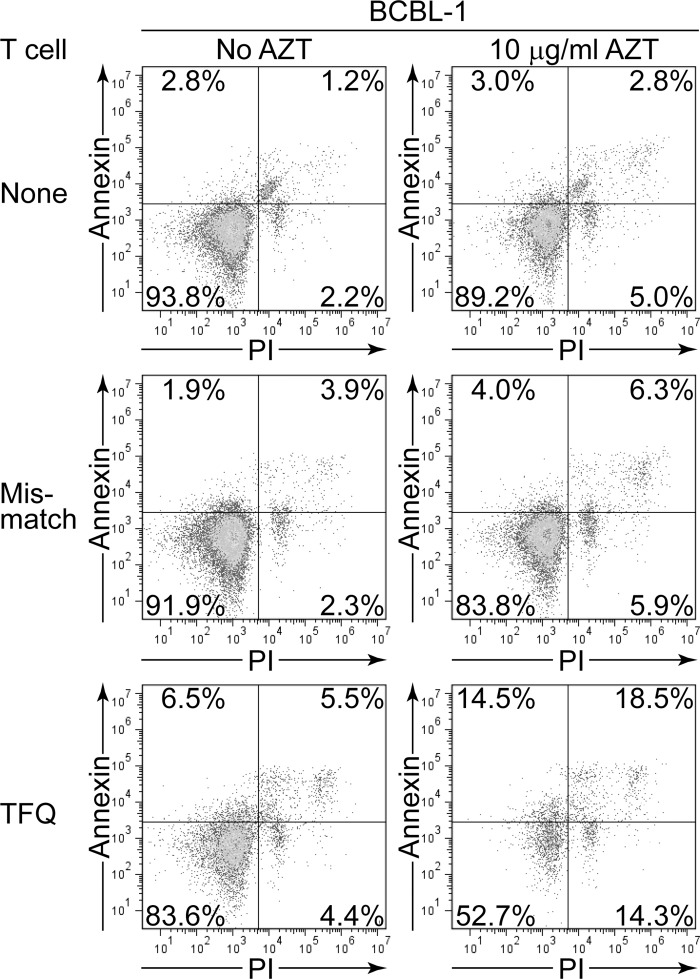
Apoptotic marker status of PELs co-cultured with KSHV-specific CD4+ T cells. BCBL-1 cells were either non-treated or cultured in 10 μg/ml AZT and challenged with MHC-matched TFQ-specific or MHC-mismatched CD4+ T cells. After five days the status of PELs binding Annexin and PI was determined by flow cytometric analysis.

To better understand the mechanism of T cell control in the outgrowth assays we examined the PELs for expression of apoptotic cell death markers during the assays. Here BCBL-1 cells were cultured either with or without AZT and parallel cultures challenged with DQ6-matched TFQ-specific or MHC-mismatched T cells. After five days PELs were stained with Annexin V to detect phosphatidylserine as a marker of apoptosis and propidium iodide (PI) to measure membrane integrity. Fig 3 shows representative flow cytometry plots of one of three assays and the percentage dead cells was estimated as the combined percentage of cells expressing Annexin V and/or PI. BCBL-1 cells cultured without T cells showed 6.2% dead cells and parallel cultures in AZT containing media showed a small increase to 10.8%. BCBL-1 cultured with MHC-mismatched T cells showed 8.1% dead cells while parallel assays conducted with AZT showed an increase to 16.2%. Assaying the MHC-matched TFQ-specific T cells against non-treated BCBL-1 showed some increase compared to the controls with 16.4% dead cells. However PELs cultured in AZT used in parallel assays with these effectors showed increased cell death with 47.3% dead cells. These studies indicate that the induction of apoptosis in the PELs correlates with the control of outgrowth of these cells.

### Expression of vFLIP and vIRF3 inhibits apoptosis from Fas and TRAIL receptor stimuli

We next sought to understand how AZT sensitized PELs to T cell control. The ability of KSHV-specific CD4+ T cells to kill targets such as Epstein-Barr virus (EBV) transformed B cells but not PELs [16] led us to hypothesize that a KSHV expressed gene was responsible for the protective effect and AZT was influencing its function. vFLIP has been shown to have anti-apoptotic function, however its low level of expression in PELs led us to examine whether other latent proteins may protect from apoptosis mediated by T cell effector mechanisms such as Fas or TRAIL receptor stimulation. Here genes encoding the PEL expressed proteins LANA, vCyclin, vFLIP, Kaposin B, vIRF3 and vIL-6 or a control empty construct were cloned into lentiviral vectors which express transgenes in a tetracycline regulated manner and BJAB cells transduced with the individual lentivirus constructs. Transgene expressing cells were then assessed for sensitivity to apoptotic killing through Fas-ligand and TRAIL receptor engagement. BJAB cells were used as they are of B cell origin and sensitive to killing through these pathways.

**Fig 4:**
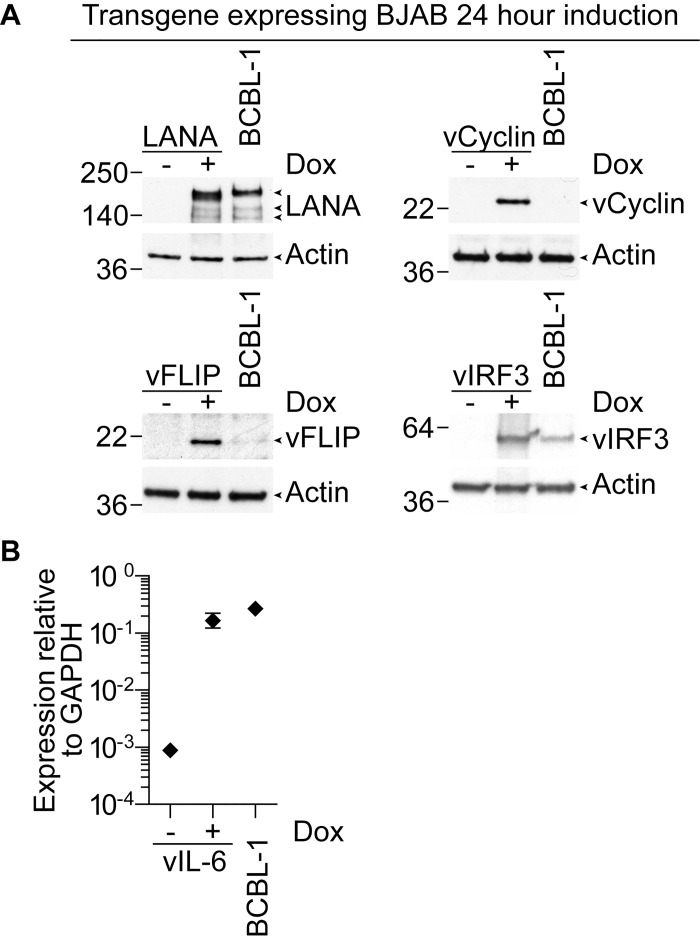
Latent KSHV transgene expression in lentivirus transduced BJAB cells. (A). Transgene expression was induced by addition of the tetracycline analogue doxycycline (Dox) to BJAB cells transduced with individual lentiviruses encoding the indicated KSHV genes. After 24 hours lysates were prepared from these and the PEL BCBL-1, for comparison, which were subjected to western blot analysis. Blots were probed with antibodies either specific to LANA, vCyclin, vFLIP or vIRF3. (B). vIL-6 expression in transduced BJAB cells was induced and after 24 hours RNA extracted from these and BCBL-1 cells. vIL-6 and GAPDH transcripts were estimated from cDNA and vIL-6 transcript abundance expressed relative to GAPDH. Error bars represent the standard error of the mean.

We first confirmed transgene expression in the BJAB cells after induction for 24 hours by western blot analysis of lysates from the cells (Fig 4A). LANA was expressed but showed a reduction in molecular weight compared to BCBL-1. Nucleotide sequence analysis indicated this construct was the same as the KSHV-BAC36 sequence from which it was derived, which in turn was established from BCBL-1. Why it has an apparent lower molecular weight in BJAB cells is unclear but may relate to differences in post translation modifications of proteins by BJAB compared to BCBL-1. vCyclin and vFLIP were expressed at markedly higher levels in transduced BJAB cells compared to BCBL-1, while vIRF3 expression was increased compared to BCBL-1. We were unable to detect protein expression of Kaposin B so no further analysis was conducted with these cells. vIL-6 protein could not be detected either in the BJAB or BCBL-1 cells, however using a previously validated qRT-PCR assay[14] we detected vIL-6 transcript at levels similar to those seen in BCBL-1 (Fig 4B).

**Fig 5:**
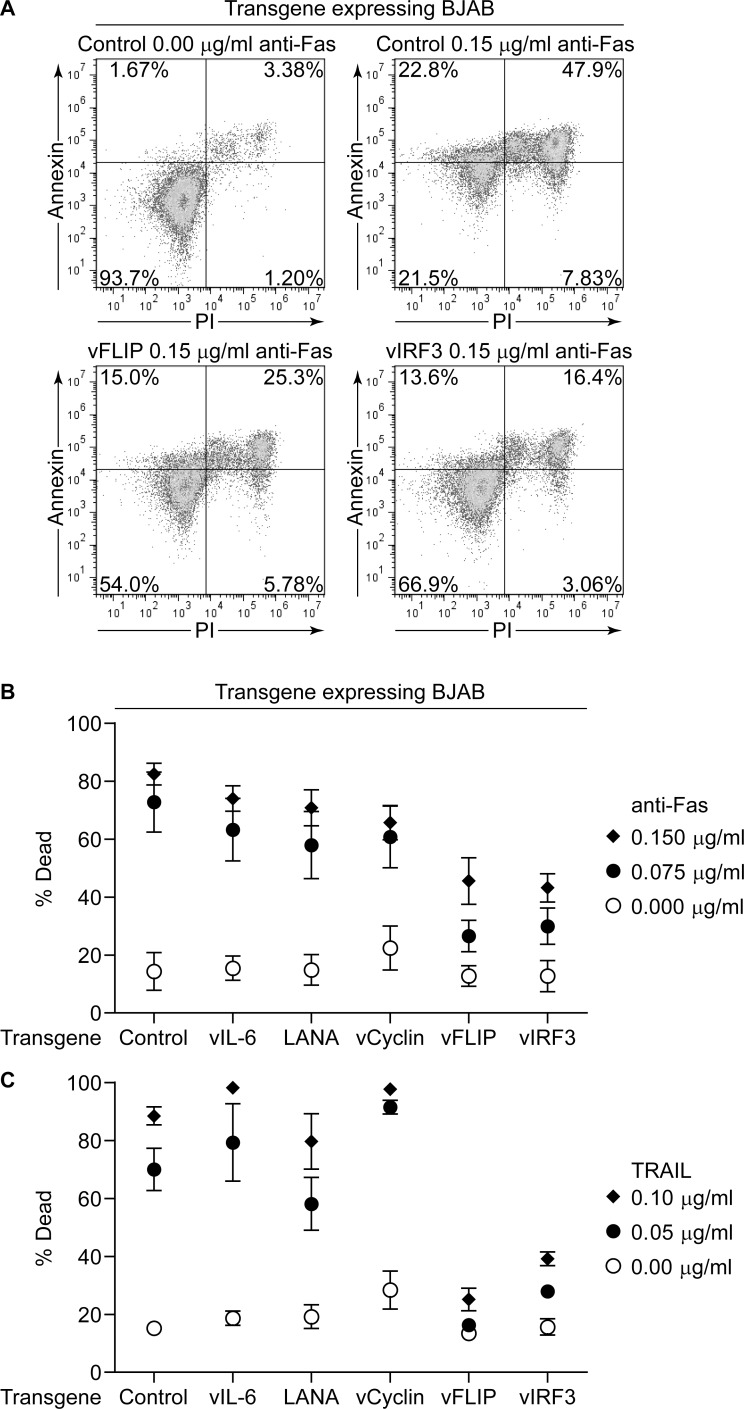
Cell death induced in BJAB cells expressing KSHV latent genes challenged with anti-Fas antibody or TRAIL for 48 hours. (A). Representative flow plots of Annexin and PI staining assays. Transgene expression in BJAB cells was induced for 24 hours and these then challenged with anti-Fas antibody or TRAIL for 48 hours. Cells were then stained with Annexin and PI, before being analyzed by flow cytometry. Representative flow plots are shown for induced BJABs with the control transgene either unchallenged or exposed to anti-Fas antibody, as well as induced vFLIP and vIRF3 transduced BJABs exposed to anti-Fas antibody. (B). Averaged results of at least five assays where transgene expressing cells were challenged with either 0.15 μg/ml or 0.075 μg/ml anti-Fas antibody and after 48 hours the percentage of dead cells binding Annexin with or without PI staining assessed (% Dead). Error bars represent standard error of the mean. (C). Averaged results of at least five assays where transgene expressing cells were challenged with either 0.1 μg/ml or 0.05 μg/ml TRAIL for 48 hours and the percentage dead cells estimated as in (B).

We next induced expression of the KSHV transgenes in the BJAB cells for 24 hours then challenged these with agonistic anti-Fas antibody or recombinant TRAIL for 48 hours, as used previously [19]. Cell death was measured as the combined percentage of cells binding Annexin V and/or staining with PI. Fig 5A shows representative FACS profiles of induced BJAB cells either unchallenged or exposed to anti-Fas antibody. Control cells showed induction of cell death upon challenge, whilst vFLIP or vIRF3 expressing BJAB showed attenuation of death. Fig 5B and 5C show averaged results from at least five independent assays of the transgene expressing cells challenged with two concentrations of anti-Fas or TRAIL. For each concentration of the apoptotic stimulus used, significant differences in mean values across the groups were tested for by one way ANOVA analysis and significant differences between groups identified by Tukey’s Honest Significant Difference test. Anti-Fas antibody induced high levels of death in control transgene expressing BJAB cells, while those expressing vIL-6, LANA and vCyclin showed no significant difference in the percentage of dead cells compared to control cells when challenged with 0.15 μg/ml or 0.075 μg/ml of anti-Fas antibody (p≥0.2248 and p≥0.1642 respectively). However BJABs expressing vFLIP or vIRF3 showed significantly reduced frequencies of dead cells after challenge with either concentration of anti-Fas antibody compared to control BJAB (vFLIP p = 5.6x10^-4^ and p = 4x10^-7^ or vIRF3 p = 2.6x10^-4^ and p = 4.1x10^-6^ for each concentration of anti-Fas). Similar challenge of control transgene expressing BJAB cells with either 0.1 μg/ml or 0.05 μg/ml TRAIL induced cell death while expression of vCyclin or vIL-6 gave no protection and LANA showed some decrease, although none of these values were significantly different to control cell death (p≥0.6164 and p≥0.3182 respectively for each concentration). However significant inhibition of cell death was again seen in vFLIP or vIRF3 expressing BJAB after challenge with either concentration of TRAIL compared to the control cells (vFLIP p<1x10^-7^ and p = 2x10^-4^ or vIRF3 p = 5x10^-7^ and p = 0.0027 for each concentration of TRAIL). These findings suggest that in addition to vFLIP, vIRF3 has the potential to inhibit apoptosis induced through Fas and TRAIL pathways.

### AZT inhibits vIRF3 mediated protection from cell death

**Fig 6:**
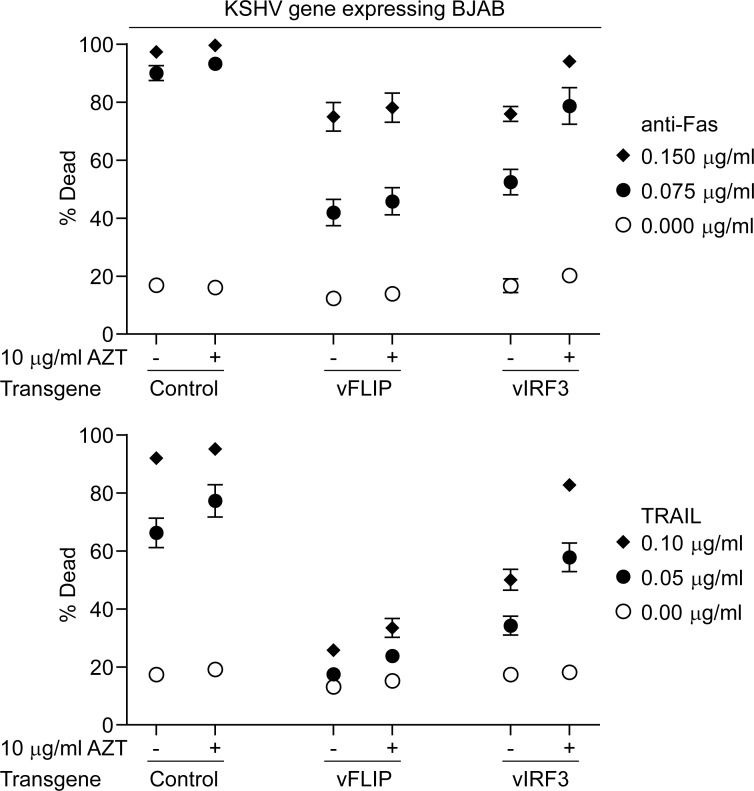
Cell death induced in BJAB cells expressing control, vFLIP or vIRF3 transgenes in the presence or absence of 10 μg/ml AZT after challenge with anti-Fas antibody or TRAIL. Averaged results of at least seven assays where control, vFLIP or vIRF3 transduced cells were grown either with or without 10 μg/ml AZT, transgene expression induced for 24 hours and cells challenged with either anti-Fas antibody or TRAIL and after 48 hours the percentage of dead cells binding Annexin with or without PI staining was then assessed.

We next determined whether AZT treatment of vFLIP or vIRF3 expressing BJAB cells restored sensitivity to anti-Fas or TRAIL challenge. Here control, vIRF3 and vFLIP BJAB cells were grown in media containing 10 μg/ml AZT for at least seven days, which did not obviously affect their growth (S3 Fig), and transgene expressing cells challenged with anti-Fas or TRAIL as before. Fig 6 shows data from at least seven independent assays; note a second more potent batch of anti-Fas antibody was used which induced higher levels of apoptosis. Compared to untreated control BJABs, AZT treated control BJABs showed non-significant increases in the percentage of dead cells when challenged with either concentration of anti-Fas (p = 0.99 and p = 0.99) or TRAIL (p = 0.94 and p = 0.41). vFLIP expressing BJABs as before showed protection upon challenge with these stimuli compared to control cells, while comparison of untreated vFLIP expressing to vFLIP expressing cells cultured in AZT showed small non-significant increases in the percentage of dead cells upon challenge with both concentrations of anti-Fas (p = 0.98 and p = 0.99) or TRAIL (p = 0.28 and p = 0.91). BJAB expressing vIRF3 again showed protection from challenge with anti-Fas or TRAIL compared to control transgene expressing cells. However parallel cultures of vIRF3-expressing BJABs treated with AZT had significantly more dead cells compared to non-treated vIRF3 expressing cells when challenged with both concentrations of anti-Fas (p = 0.0034 and p = 0.0014) or TRAIL (p<1x10^-7^ and p = 0.0027). These results indicate that in this system, AZT inhibits vIRF3 mediated protection from anti-Fas and TRAIL challenge.

**Fig 7:**
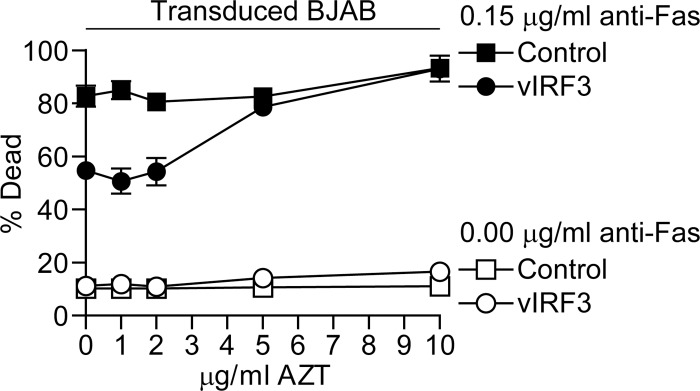
Titration of AZT inhibition of vIRF3 function in BJAB cells. vIRF3 and control transduced BJAB cells were cultured in different concentrations of AZT and then transgene expression induced after which the cells were challenged with anti-Fas antibody and the percentage dead cells determined as in Fig 5. Results are averages of three independent assays with error bars representing the standard error of the mean.

To probe the effectiveness of AZT inhibition of vIRF3 protection from apoptotic challenge, we titrated the concentration of AZT that the vIRF3 and control transgene expressing BJAB cells were cultured in and then challenged these cells with anti-Fas antibody. Fig 7 shows compiled results of three assays measuring the percentage of dead cells 48 hours after challenge. As before, vIRF3 expressing cells treated with 10 μg/ml AZT lost their protection from apoptosis induction as did those treated with 5 μg/ml AZT. However concentrations of AZT at 2 μg/ml or less gave no inhibition of vIRF3 protection.

### AZT treatment interferes with other vIRF3 phenotypes in PELs

**Fig 8:**
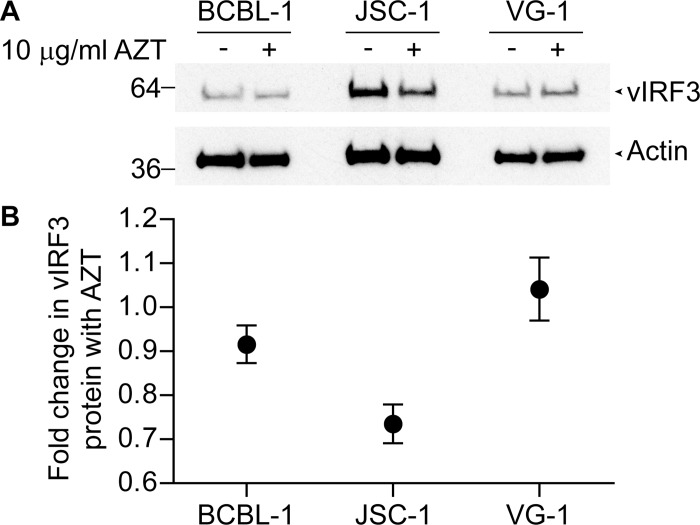
vIRF3 levels in AZT treated PEL lines. A. BCBL-1, JSC-1 and VG-1 PELs were either treated with 10 μg/ml AZT or not, lysates made from the lines and subjected to western blot analysis, probing for vIRF3 or actin expression. One representative blot of three is shown. B. Average fold change of vIRF3 expression was determined by estimating relative levels of vIRF3 compared to actin in pairs of PELs either treated with or without 10 μg/ml AZT and the ratio of vIRF3 expression between the pairs determined. Error bars represent the standard error of the mean.

The ability of AZT to inhibit vIRF3 protective function led us to examine whether AZT treatment altered expression of this protein in the PEL lines. Here lysates from PELs which had been used in outgrowth assays and either treated with 10 μg/ml AZT or not were made and subjected to western blot analysis for vIRF3 levels. Fig 8A displays one representative blot of three, showing vIRF3 or actin levels in BCBL-1, JSC-1 and VG-1 cells while Fig 8B shows compiled fold change in vIRF3 expression from the three assays. AZT treatment of BCBL-1 and VG-1 induced small if any changes in vIRF3 levels while some decreased expression was seen in AZT treated JSC-1 cells. These results indicate that AZT treatment can reduce vIRF3 protein levels in some PEL lines but not others, suggesting its mechanism of action may be through reduction of vIRF3 levels in some lines but potentially through inhibiting vIRF3 function in others.

**Fig 9:**
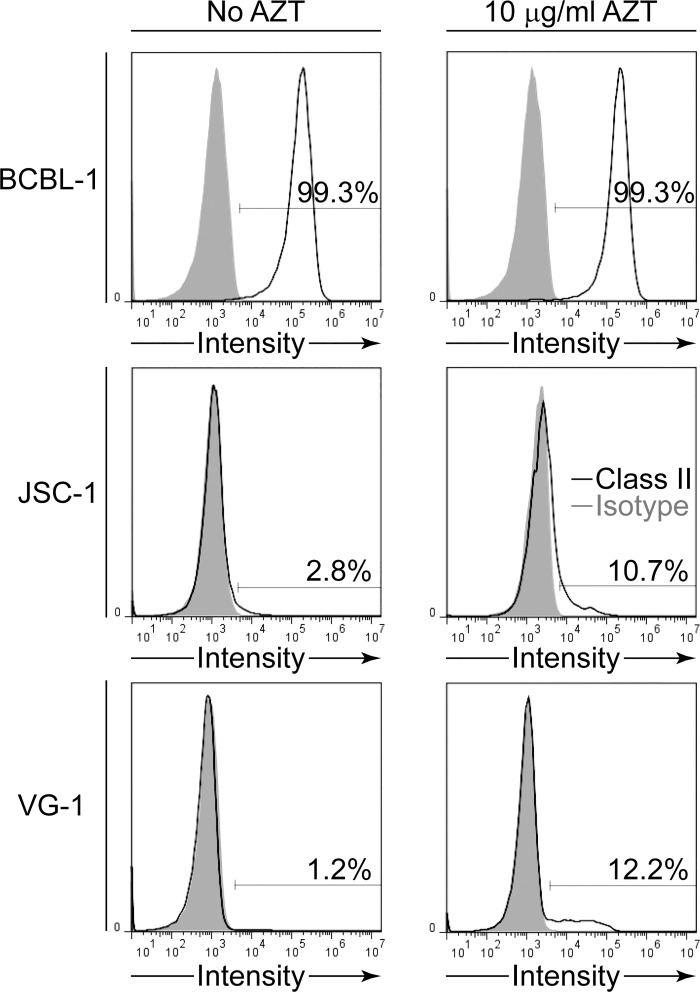
Surface expression of MHC class II on PELs treated with AZT. BCBL-1, JSC-1 or VG-1 PEL cells were cultured in either normal medium (No AZT) or medium supplemented with 10 μg/ml AZT for at least 10 days, then stained with MHC class II-specific antibodies or an isotype control and analyzed by flow cytometry. Data is representative of three assays.

We finally investigated whether AZT interfered with other vIRF3 functions by examining a second phenotype associated with its expression, namely modulation of surface MHC class II expression on PEL cells [12]. As shown in Fig 9 treatment of BCBL-1 with AZT, which constitutively expresses high levels of surface MHC class II [16], did not alter expression. However the PELs JSC-1 and VG-1, which constitutively express low levels of surface class II, when treated with AZT showed increased proportions of cells expressing MHC class II. These studies are consistent with AZT treatment interfering with vIRF3 function.

## Discussion

This work was prompted by our observations that although KSHV-specific CD4+ T cells could recognize PELs expressing MHC class II [17] or PELs engineered to express surface class II, they were unable to control them despite killing other cell types [16]. To make PELs more sensitive to T cell control, we treated PELs with AZT and found that LANA- and vCyclin-specific CD4+ T cells inhibited or completely controlled the outgrowth of PELs in co-culture assays. The inhibition observed in these assays was equivalent or more efficient than that seen in similar assays using CD4+ T cells specific for latent epitopes from the related human γ-herpesvirus EBV to control outgrowth of EBV-transformed B cells [26].

To identify how AZT induced sensitivity to T cell control we hypothesized that it was interfering with pro-survival KSHV-gene functions which inhibit cell death pathways initiated by T cell recognition, namely those of the extrinsic apoptotic pathway. Such KSHV gene products would include vFLIP which can inhibit killing via Fas [27,28] and a second receptor mediated killing mechanism, TNF-α [29]. Consistent with these studies we found that expression of vFLIP in BJAB cells inhibited killing by anti-Fas antibody and TRAIL. Unexpectedly we found that expression of vIRF3 also inhibited apoptosis induced by these two mechanisms. vIRF3 is a polyfunctional protein which subverts numerous host pathways, some of which are linked to apoptosis. Thus knockdown of vIRF3 in PELs induces growth arrest, caspase 3 activation and apoptosis [30]. vIRF3 has further been reported to bind and inhibit p53 function [10], and bind to the forkhead box class O (FoxO) 3a transcription factor and its regulatory 14-3-3 proteins. This causes retention of FoxO factors in the cytoplasm, preventing their ability to induce expression of proapoptotic proteins such as Bim [31]. However these mechanisms would seem unlikely to intersect with the extrinsic apoptotic pathways triggered in our study. vIRF3 has also been shown to interfere with apoptosis induced by overexpression of the interferon induced dsRNA-activated protein kinase R [32], which is known to require the FADD/caspase pathway for apoptosis induction. However it is not thought that interactions through receptors such as Fas are responsible for cell death induction in this situation [33]. Our study suggests that vIRF3 inhibits the extrinsic apoptotic pathway using a different mechanism to those so far described.

The ability of vFLIP and vIRF3 to protect BJAB cells from extrinsic apoptotic stimuli led us to examine whether AZT treatment restored sensitivity to these stimuli and we found that while vFLIP expressing BJAB remained resistant, vIRF3 expressing cells became sensitive. This result was surprising as vFLIPs pro-survival function is thought to be mediated through its ability to activate NF-κB through the canonical pathway by interacting with the IκB kinase (IKK) complex [34] and AZT is believed to inhibit IKK mediated phosphorylation of IκB [21]. One may have predicted then that AZT would affect vFLIP function, however vFLIP can also activate the alternative NF-κB pathway [35] and whether AZT can inhibit this pathway is unknown. Regardless of this, the current experiments indicate that AZT can affect pathways manipulated by KSHV in addition to NF-κB. The inhibition of vIRF3 mediated protection from apoptosis by AZT suggests that either AZT alters a cellular pathway that vIRF3 interacts with, or AZT may act more directly on vIRF3 itself. Support for the latter possibility comes from our observations that culturing the low class II expressing JSC-1 and VG-1 PELs in AZT increased surface MHC class II expression on a subset of cells. This finding is consistent with the drug inhibiting vIRF3 function, preventing repression of CIITA expression, restoring some degree of MHC class II transcription [12]. Additionally in this context, we believe it is unlikely that increased expression of surface class II itself is sufficient for T cell control of PEL outgrowth as BCBL-1 cells express relatively high levels of surface class II and can be recognized by CD4+ T cells [16], yet in the absence of AZT are not controlled by the T cells. Furthermore we have previously found that inducing expression of CIITA in PELs, which consequently upregulates surface class II expression, allows recognition by KSHV-specific CD4+ T cells but not killing of these tumor cells [16].

Although we have demonstrated a role for AZT in inhibiting vIRF3 function, we can not exclude that this drug also alters function of the CD4+ T cell clones used in our assays. However murine T cells treated with similar concentrations of AZT maintain their cytotoxic function when cultured in AZT containing medium [36]. Similarly human PBMC maintained in lower concentrations of AZT compared to the present study also show no obvious difference in cytokine secretion after stimulation with phytohaemagglutanin or anti-CD3 [37]. Nevertheless AZT treatment of PBMC is known to be toxic to mitochondria within T cells or induce apoptosis in these cells [38], and may reduce their proliferative potential after stimulation with anti-CD3 [39]. However in the outgrowth assays conducted here we would expect to see little proliferation of the T cells in the cytokine poor environment of these experiments.

The protection afforded by vFLIP and vIRF3 in BJAB cells upon anti-Fas or TRAIL challenge suggests both may contribute to protection from such challenges in PELs and potentially, inhibition of one may be compensated for by the other. Assigning the contribution of either viral product to protection from apoptotic stimuli may be assessed through using CRISPR/Cas9 mediated genome editing to establish PEL lines lacking either of these genes, as used to manipulate other herpesvirus infected cells [40]. However knock down of either vFLIP or vIRF3 expression in PELs sensitizes or induces apoptosis in these cells, so establishing lines deficient in either of these may not be possible [18,30]. Such a question may be best addressed by making viruses lacking either or both of these genes, using these to infect B cell lines which are known to support virus expression of vIRF3, such as BJAB or primary B cells [14,41], and then assess apoptotic sensitivity of these cells. Nevertheless, inhibiting function of either of these may be sufficient then to allow improved control by immune effectors. Thus even though AZT only inhibited vIRF3 function in the BJAB model, inhibition mediated by AZT over vIRF3 in PELs may be sufficient to induce their sensitization to CD4+ T cell control.

Recently AZT has been used successfully with valganciclovir in an MCD setting where these prodrugs are thought to be phosphorylated to toxic compounds by KSHVs lytic cycle expressed thymidine kinase (ORF21) and phosphotransferase (ORF36) respectively[42]. Infected B lymphocytes within MCD lesions also express vIRF3 [10] and potentially AZT may be inhibiting this proteins function, allowing better targeting by KSHV-specific CD4+ T cells. Important in this context is that MCD patients, who are usually co-infected with HIV, have relatively preserved immune competence in terms of CD4+ T cell numbers [42,43], although no studies have so far examined KSHV-specific CD4+ T cell immunity in this patient group. Correlating KSHV-specific CD4+ T cell responses with patient outcome upon AZT treatment would help give an understanding of whether this drug and immune effector combination is capable of controlling KSHV-infected cells in this group. Additionally, little is known as to when vIRF3 is expressed in vivo, outside of cases of MCD and PEL. However as the gene appears to be expressed in infected B lymphocytes [13,14,41] it would seem likely that it is expressed during the establishment and maintenance of the latent KSHV reservoir in B lymphocytes. Potentially then, AZT treatment may help make infected B lymphocytes within the latent reservoir more susceptible to T cell control. However our studies using the BJAB-vIRF3 transduced cells indicates that a concentration of 5 μg/ml AZT is required to inhibit vIRF3 function which would likely exceed plasma levels attained after a standard oral dose of 200 mg of AZT that results in plasma AZT levels of 0.9–2.9 μg/ml [44]. To attain sufficient levels it may be necessary to use high doses like those used in the above MCD setting of multiple oral doses of 600 mg of AZT [42]. Nevertheless our studies suggest that interfering with vIRF3 function may help restore the virus-host balance.

## Materials and Methods

### Cell lines and outgrowth assays

CD4+ T cell clones were established in a previous study [16] and their characteristics are shown in Table 1. An additional specificity was included which recognized the HLA-DQ*0601 presented vCyclin encoded peptide TFQQSLTSHMRKLLG. PEL cells were maintained in RPMI-1640 (Sigma-Aldrich) with 10% fetal calf serum (FCS) (LifeTechnologies). PELs used in outgrowth assays were VG-1 (a kind gift from Prof Christian Brander, IrsiCaixa AIDS Research Institute–HIVACAT, Barcelona, Spain [45]), BCBL-1 (NIH AIDS Reagent Program, catalogue number 3233, [46]) and JSC-1 (American Type Culture Collection, catalogue number CRL-2769, [47]). Outgrowth assays were performed by seeding doubling dilutions of triplicate PEL cell cultures in 96 well plates, ranging from concentrations of 10 000 to 1250 cells per well. PEL cultures had 10 000 CD4+ T cells added and assays were conducted in the presence or absence of 10 μg/ml AZT (Sigma-Aldrich). Cultures were incubated for 10 days, after which cell outgrowth identity was determined by staining cells with antibodies specific to CD138 or CD4 (Biolegend). Stained cells were analyzed on an Accuri C6 flow cytometer (BD Biosciences) and data analyzed using Flowjo (Treestar). Outgrowth assays were scored as the minimal number of PELs seeded to overgrow T cells, as defined by there being an equal or greater frequency of PEL cells compared to T cells at the end of the assay. Surface MHC class II expression on PEL lines was measured by staining with antibodies specific to HLA-DR (L243, Biolegend) and analyzing by flow cytometry.

### qRT-PCR detection of Fas-ligand, TRAIL and KSHV transcripts

CD4+ T cell clones were activated by stimulation with peptide-epitope sensitized Epstein-Barr virus-transformed B cells for five hours, RNA extracted and cDNA synthesized as described[14]. Fas-ligand and TRAIL transcript levels were assessed by Taqman Gene Expression Assays (ThermoFisher) on 5 ng of cDNA as described by the manufacturer. Relative levels of transcripts were determined by comparing to dilutions of cDNA prepared from peripheral blood mononuclear cells (PBMC) activated with phorbol myristate acetate and ionomycin for 5 hours[24,25].

KSHV transcript levels were estimated on RNA extracted from PELs using validated qRT-PCR assays as previously described [14].

### Ethics statement

PBMC were collected from healthy donors after obtaining written informed consent to donate samples and experiments were approved by the South Birmingham Research Ethics Committee (approval reference number 06/Q2707/300).

### Production and characterization of lentiviruses

The KSHV latent genes vCyclin, vFLIP, Kaposin B, vIRF3 and vIL-6 were PCR amplified from cDNA extracted from the BCBL-1 PEL, additionally Kaposin B was epitope tagged with the influenza HA epitope. Sequence encoding LANA was excised from the BCBL-1 derived BAC36 construct [48] and these genes and a control vector lacking an insert cloned individually into pENTR1a (ThermoFisher). Constructs were recombined into the lentiviral vector pInducer 20[49], which expresses transgenes under the control of a tetracycline regulated promoter and the resulting plasmid co-transfected with standard plasmids encoding lentiviral packaging and envelope genes into 293T cells (American Type Culture Collection, catalogue number CRL-3216). After 48 hours, supernatants were used to infect BJAB cells (a kind gift from Prof George Klein [50]) and transduced BJABs selected by culturing in RPMI-1640 10% FCS supplemented with 200 μg/ml G418. Transgene expression was assessed by inducing expression with 2 μg/ml doxycycline for 24 hours, then lysing cells for either western blot or qRT-PCR analysis as previously described[14,16]. Blots were probed with antibodies specific to LANA (clone LN-53, Advanced Biotechnologies), vCyclin (94B, Abcam), vFLIP (4C1)[51], HA (3F10, Roche), vIRF3 (CM-A807, Abnova), vIL-6 (Abbiotec), and actin (AC-74, Sigma) and these detected using an appropriate anti-species HRP-conjugated antibody, followed by ECL detection (GE Healthcare).

### Apoptosis assays

Transgene expression in BJAB cells was induced for 24 hours by addition of 2 μg/ml doxycycline, after which 150 000 cells were incubated in 100 μl of media with the indicated concentrations of either an agonistic anti-Fas antibody (CH11, Beckman-Coulter) or recombinant TRAIL (Peprotech) for 48 hours. Cell death was estimated using an Annexin V-propidium iodide apoptosis detection kit (eBioscience) and cells analyzed by flow cytometry.

### Statistical analysis

Statistical comparisons between levels of cell death in apoptosis assays were performed using the R statistical program (v 3.0.2, R Development Core Team, http://www.R-project.org). So that parametric statistical tests could be used we first assessed whether the data satisfied the assumptions for use of these statistics namely that it was normally distributed using the Shapiro-Wilk test, and was homoscedastic using Levene’s test. To identify whether there were variation in mean levels of cell death between groups, these were tested by one way ANOVA and groups which differed were identified by post-hoc testing with Tukey’s Honest Significant Difference test.

## Supporting Information

S1 Fig KSHV gene transcript levels and proliferation of PELs treated with 10 μg/ml AZT. A. RNA was extracted from parallel cultures of PELs either untreated or treated with 10 μg/ml AZT. cDNA was subjected to qRT-PCR analysis for either the tricistronic LANA-vCyclin-vFLIP, bicistronic vCyclin-vFLIP, monocistronic vFLIP, vIL-6, vIRF3, ORF50 and GAPDH transcripts. Transcript levels are expressed relative to GAPDH abundance. Error bars represent standard error of the mean. B. PELs were seeded in 96 well U bottom plates in replicates of 10 000 cells in 200 μl of media supplemented with or without 10 μg/ml AZT. Cells were counted over a seven day period and representative results of one of two assays are shown. Error bars indicate standard error of the mean.
(TIF)

S2 Fig PEL outgrowth assays conducted using KSHV-specific CD4+ T cells pre-treated with AZT. A. VG-1 PELs treated with 10 μg/ml AZT or not were seeded in triplicate cultures at doubling dilutions from 10^4^ cells per well to 1 250 cells per well. To these, 10^4^ MHC-matched LAP/LRS -specific CD4+ T cells, either clone 112 or clone 146 were added, or mismatched CD4+ T cell clones were added, namely clone 29 or clone 24. In parallel, cultures were established which used T cell clones which had been pre-treated with 10 μg/ml AZT for four days. Where indicated AZT was added to a final concentration of 10 μg/ml in the cultures. Cell mixtures were allowed to grow for 10 days after which cell outgrowth was scored. Results are expressed as the minimum number of PELs seeded which successfully outgrew the T cells and the dashed line represents the number of AZT treated PELs seeded in the absence of T cells to achieve outgrowth. Black arrowheads indicate greater than 10^4^ PELs were required to outgrow the T cells. B. Outgrowth assays were set up as in A but using BCBL-1 cells which were challenged with TFQ-specific CD4+ T cell clones 1 or 2. Results shown are representative of one of two assays.
(TIF)

S3 Fig Proliferation of BJAB cells treated with 10 μg/ml AZT. BJAB cells transduced with the control lentivirus were seeded in 96 well U bottom plates in replicates of 10 000 cells in 200 μl of media supplemented with or without 10 μg/ml AZT. Cells were counted over a seven day period and representative results of one of two assays are shown. Error bars indicate standard error of the mean.
(TIF)
