# Supplementary figures and images for: Azidothymidine Sensitizes Primary Effusion Lymphoma Cells to Kaposi Sarcoma-Associated Herpesvirus-Specific CD4+ T Cell Control and Inhibits vIRF3 Function
Source: PLoS Pathog. 2016 Nov 28;12(11):e1006042. doi: 10.1371/journal.ppat.1006042 (PMC5125715; doi:10.1371/journal.ppat.1006042)

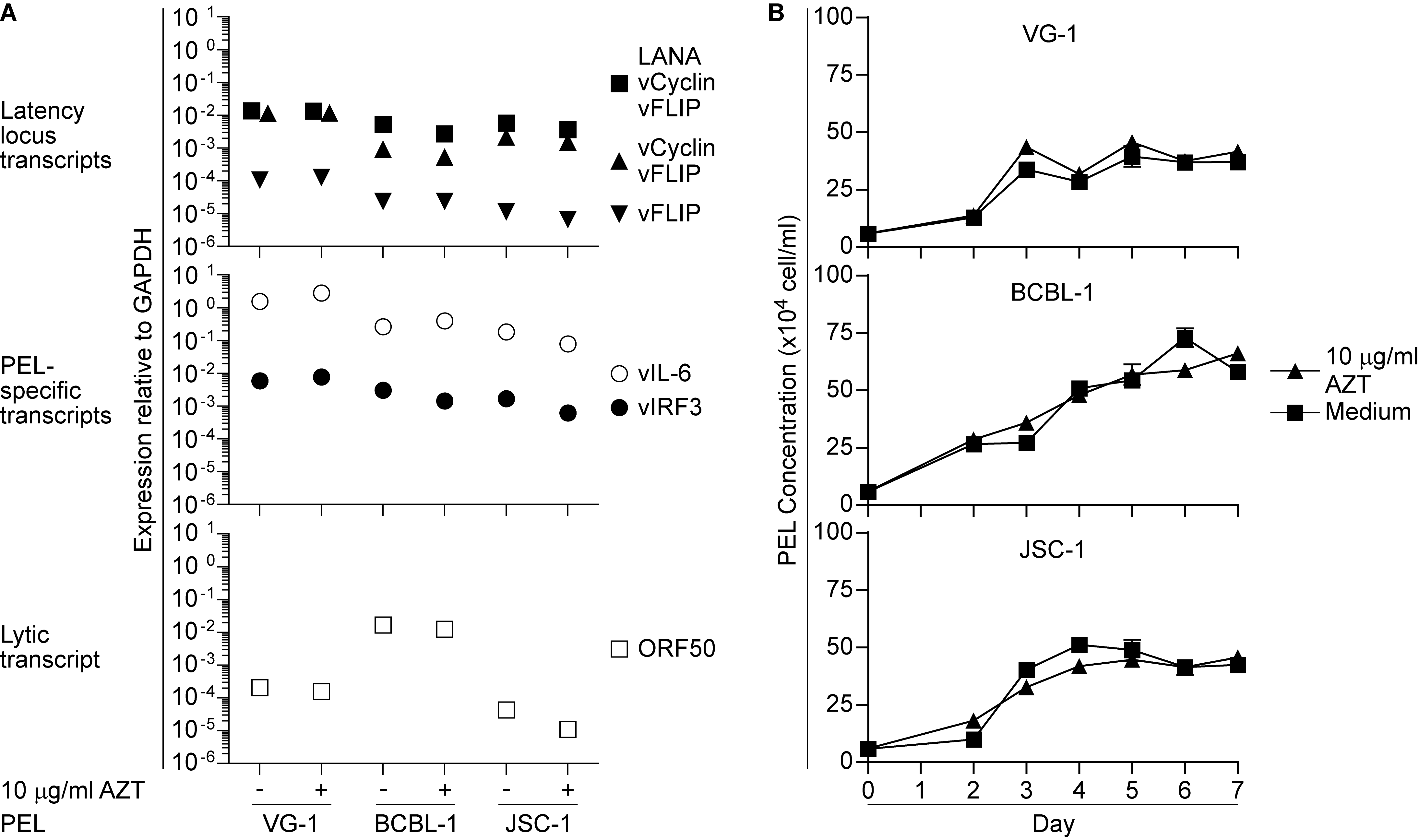

Supplement: S1 Fig — A. RNA was extracted from parallel cultures of PELs either untreated or treated with 10 μg/ml AZT. cDNA was subjected to qRT-PCR analysis for either the tricistronic LANA-vCyclin-vFLIP, bicistronic vCyclin-vFLIP, monocistronic vFLIP, vIL-6, vIRF3, ORF50 and GAPDH transcripts. Transcript levels are expressed relative to GAPDH abundance. Error bars represent standard error of the mean. B. PELs were seeded in 96 well U bottom plates in replicates of 10 000 cells in 200 μl of media supplemented with or without 10 μg/ml AZT. Cells were counted over a seven day period and representative results of one of two assays are shown. Error bars indicate standard error of the mean. (TIF) [file ppat.1006042.s001.tif]

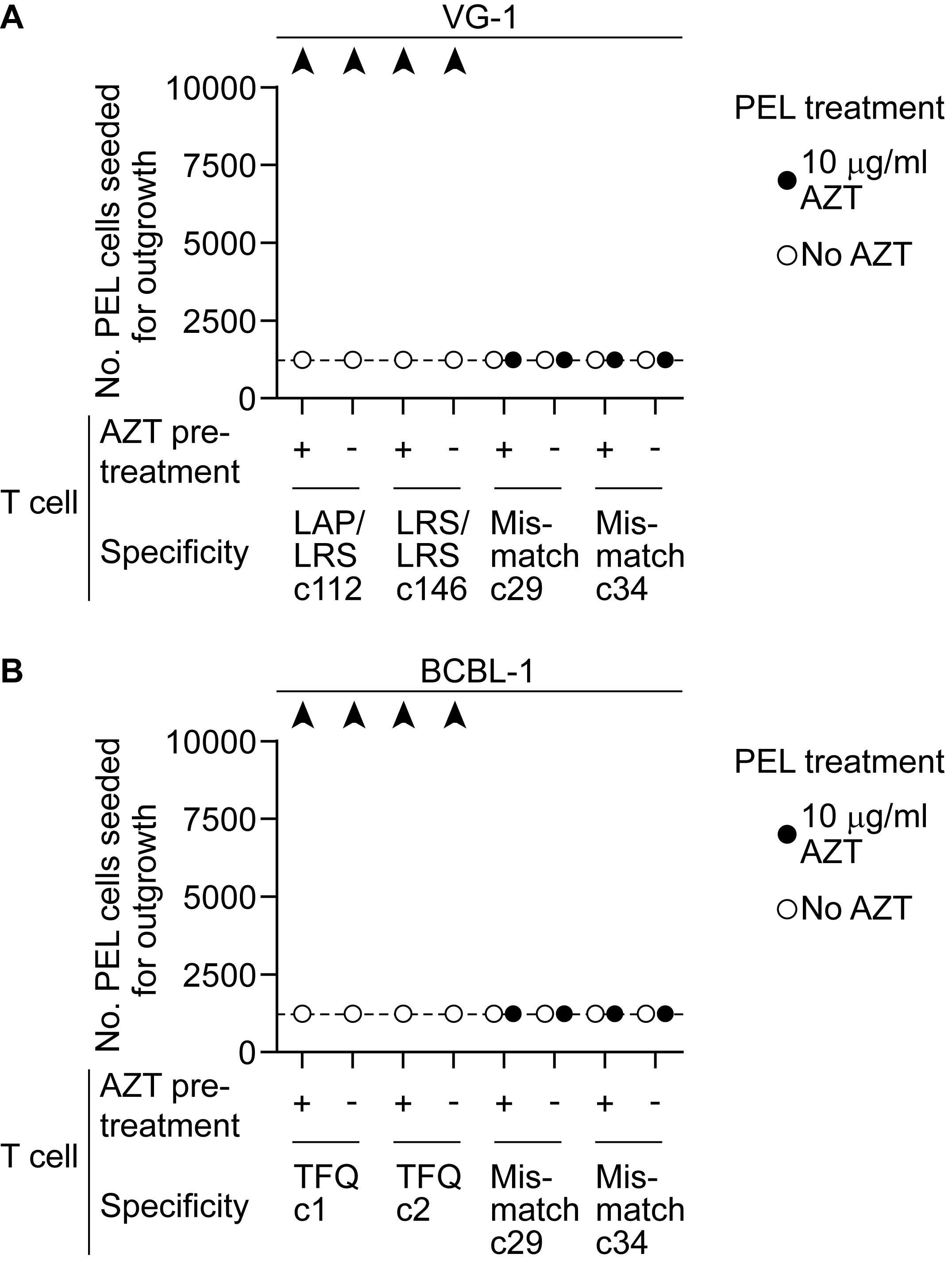

Supplement: S2 Fig — A. VG-1 PELs treated with 10 μg/ml AZT or not were seeded in triplicate cultures at doubling dilutions from 104 cells per well to 1 250 cells per well. To these, 104 MHC-matched LAP/LRS -specific CD4+ T cells, either clone 112 or clone 146 were added, or mismatched CD4+ T cell clones were added, namely clone 29 or clone 24. In parallel, cultures were established which used T cell clones which had been pre-treated with 10 μg/ml AZT for four days. Where indicated AZT was added to a final concentration of 10 μg/ml in the cultures. Cell mixtures were allowed to grow for 10 days after which cell outgrowth was scored. Results are expressed as the minimum number of PELs seeded which successfully outgrew the T cells and the dashed line represents the number of AZT treated PELs seeded in the absence of T cells to achieve outgrowth. Black arrowheads indicate greater than 104 PELs were required to outgrow the T cells. B. Outgrowth assays were set up as in A but using BCBL-1 cells which were challenged with TFQ-specific CD4+ T cell clones 1 or 2. Results shown are representative of one of two assays. (TIF) [file ppat.1006042.s002.tif]

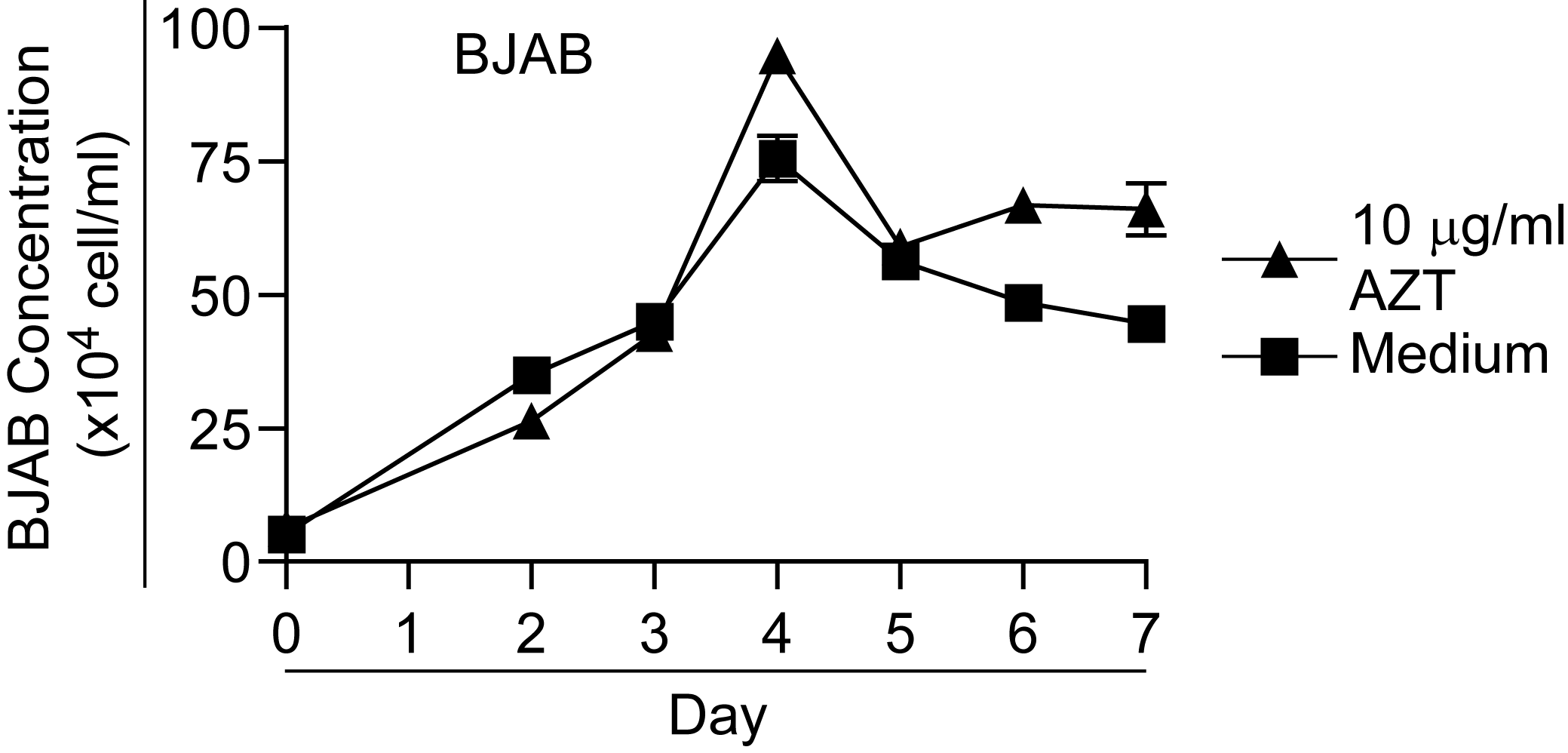

Supplement: S3 Fig — BJAB cells transduced with the control lentivirus were seeded in 96 well U bottom plates in replicates of 10 000 cells in 200 μl of media supplemented with or without 10 μg/ml AZT. Cells were counted over a seven day period and representative results of one of two assays are shown. Error bars indicate standard error of the mean. (TIF) [file ppat.1006042.s003.tif]
